# Parasite co-infections show synergistic and antagonistic interactions on growth performance of East African zebu cattle under one year

**DOI:** 10.1017/S0031182013001261

**Published:** 2013-09-04

**Authors:** S. M. THUMBI, B. M. de. C. BRONSVOORT, E. J. POOLE, H. KIARA, P. TOYE, M. NDILA, I. CONRADIE, A. JENNINGS, I. G. HANDEL, J. A. W. COETZER, O. HANOTTE, M. E. J. WOOLHOUSE

**Affiliations:** 1 Centre for Infectious Diseases, University of Edinburgh, Ashworth Laboratories, Kings Buildings, West Mains Road, Edinburgh EH9 3JT, UK; 2 The Roslin Institute, Easter Bush, University of Edinburgh, Roslin, Midlothian EH25 9RG, UK; 3 International Livestock Research Institute, P.O. Box 30709, Nairobi 00100, Kenya; 4 Department of Veterinary Tropical Diseases, Faculty of Veterinary Science, University of Pretoria, Private bag X04, Onderstepoort, South Africa; 5 School of Life Science, University of Nottingham, University Park, Nottingham NG7 2RD, UK

**Keywords:** co-infections, interactions, growth rate, cattle

## Abstract

The co-occurrence of different pathogen species and their simultaneous infection of hosts are common, and may affect host health outcomes. Co-infecting pathogens may interact synergistically (harming the host more) or antagonistically (harming the host less) compared with single infections. Here we have tested associations of infections and their co-infections with variation in growth rate using a subset of 455 animals of the Infectious Diseases of East Africa Livestock (IDEAL) cohort study surviving to one year. Data on live body weight, infections with helminth parasites and haemoparasites were collected every 5 weeks during the first year of life. Growth of zebu cattle during the first year of life was best described by a linear growth function. A large variation in daily weight gain with a range of 0·03–0·34 kg, and a mean of 0·135 kg (0·124, 0·146; 95% CI) was observed. After controlling for other significant covariates in mixed effects statistical models, the results revealed synergistic interactions (lower growth rates) with *Theileria parva* and *Anaplasma marginale* co-infections, and antagonistic interactions (relatively higher growth rates) with *T. parva* and *Theileria mutans* co-infections, compared with infections with *T. parva* only. Additionally, helminth infections can have a strong negative effect on the growth rates but this is burden-dependent, accounting for up to 30% decrease in growth rate in heavily infected animals. These findings present evidence of pathogen–pathogen interactions affecting host growth, and we discuss possible mechanisms that may explain observed directions of interactions as well as possible modifications to disease control strategies when co-infections are present.

## INTRODUCTION

Events that occur early in a host's life, including infection with pathogens, are important determinants of the reproductive and production success of individuals. Parasite infections cause significant losses through loss of body condition, inefficiency of food utilization, for example in helminth infections, decreased reproductive fitness or deaths, and are a major threat to success of infected individuals. Infections in natural populations are known to involve multiple pathogens rarely occurring as single infections (Boag *et al*. 2001; Cox, 2001; Lello and Hussell, 2008). Despite this, little is known about the consequences of harbouring co-infections on important traits such as growth rates, and whether there are pathogen–pathogen interactions that should be considered in programmes aimed at disease control.

Within a host, the co-infecting pathogens may not always act independently of each other, and may interact, modifying the densities of each other and their impact on the infected host as opposed to when they exist as single infections (Craig *et al*. 2008; Telfer *et al*. 2010). Various mechanisms for these interactions have been suggested including community ecology theories as competitive interactions between pathogens sharing similar resources or location in a host (Pedersen and Fenton, 2007; Graham, 2008) or interactions with host immune system where new parasites infecting a host find an immuno-environment created in response to previous or current infections (Lafferty, 2010; Telfer *et al*. 2010). Such interactions complicate current disease-by-disease impact studies and accurate estimates of disease burden would need to account for the impact that parasite–parasite and parasite–host interactions have on host outcomes (Fenton and Perkins, 2010).

Here we study co-infections and their impact on East African zebu cattle, which are raised in smallholder production systems under low veterinary input for disease control and treatment. The environment they are raised in is endemic for a range of micro- and macro-parasites, and provides a good system to study co-infections and increase our understanding of parasite interactions and their impact on host outcomes. Zebu cattle are an important source of livelihood for a large rural population in sub-Saharan Africa, and their improved production present opportunities for improving the livelihoods of these livestock keepers (Perry, 2002, 2007; Kristjanson *et al*. 2004; Tarawali *et al*. 2011). Despite zebus’ relative resistance and resilience compared with European breeds (Ndungu *et al*. 2005), livestock diseases in zebu cattle remain a major constraint causing mortalities and sub-optimal production, and their control is seen as an important step towards improved production and better livelihoods (Perry, 2002, 2007; Tomley and Shirley, 2009).

By following a cohort of indigenous zebu cattle raised in disease-endemic areas in Western Kenya, here we study the weight–age relationship from birth to one year. Specifically, we investigate: (a) the impact of non-infectious factors on the growth rate, and controlling for these non-infectious factors; (b) the effect of individual infections on growth rate of calves less than one year; and (c) the impact of different co-infection profiles on growth rates. This study seeks to evaluate the costs of an animal surviving parasitic infections by determining the differential impact that infections, co-infections and their interactions have on the outcome ‘growth rate’. Knowledge of environmental factors, infections and co-infection profiles with the greatest impact on growth rates should be an integral part of design programmes aimed at disease control, including improved livestock production.

## MATERIALS AND METHODS

### Data collected

Between October 2007 and September 2010, a total of 548 East African zebu calves were recruited into the Infectious Diseases of East African Livestock (IDEAL) cohort study at birth and followed during their first year of life. The animals came from 20 sub-locations (smallest administrative units) falling within four agro-ecological zones in Western Kenya. Each study animal was routinely monitored every 5 weeks from birth until one year or earlier if lost from the study. During the follow-up period, routine clinical examinations on study calves were conducted every 5 weeks, and blood, fecal and other clinically relevant samples collected, stored and later processed for laboratory diagnosis. Data on farm management practices, herd health and veterinary interventions on the herd during the inter-visit period were collected. Live body weight (kg) and girth measurements (cm) were recorded at recruitment, and every 5 weeks thereafter until 31 weeks old. A final body weight and girth measurement was taken at 51 weeks before animals left the study. Additionally, maternal data including the dam's general health, udder health, girth measurements and body condition score were recorded during each corresponding calf visit, until weaning or the calf left the study. Detailed description of the study design and protocol are provided by Bronsvoort *et al*. (in press). In this study we use data on a subset of 455 calves of the IDEAL cohort that completed a full year of observation. The study received approval by the University of Edinburgh Ethics Committee (reference number OS 03–06), and the Animal Care and Use Committee of the International Livestock Research Institute. All participating farmers gave informed consent in their native language before recruiting their animals into the study.

### Predictor variables

Variables that were tested for their association with growth of calves during the first year of life included: (a) Farmer-related factors: including farmer's education level, main occupation, gender and age, as well as herd and land sizes owned; (b) management factors: including livestock housing, disease, feeding and watering, and disease control practices within the farm; (c) calf factors: calf sex, level of European taurine introgression and heterozygosity; (d) environmental factors: elevation, Normalized Difference Vegetation Index (NDVI); and (e) dam factors: body condition scores, heart girth measurements, general health and udder health.

### Infection data

Fecal, blood and other clinically relevant samples such as skin scrapings and bacterial swabs collected during the monitoring visits were processed and screened for a range of micro- and macro-parasites. The pathogens screened for and diagnostic methods used are provided in Table 1.

**Table 1.** Summary table showing different samples collected, data type, pathogens screened for and the diagnostic tests used

Sample	Diagnostic test	Data type	Pathogens	Reference
Blood smears	Microscopy (Giemsa stain)	Binary	*Anaplasma* spp., *Babesia* spp., *Theileria* spp., *Trypanosoma* spp.	(OIE, 2008)
Lymph node smears	Microscopy (Giemsa stain)	Binary	*Theileria* spp., *Trypanosoma* spp.	(OIE, 2008)
EDTA blood	Haematocrit Centrifugation	Binary	*Trypanosoma* spp.	(Woo, 1970)
EDTA blood	Dark ground microscopy	Binary	*Trypanosoma* spp.	(Murray *et al*. 1977)
EDTA blood	Serology – ELISA	Binary	*Anaplasma marginale, Babesia bigemina, Theileria mutans, Theileria parva*	(Katende *et al*. 1998; Morzaria *et al*. 1999; Tebele *et al*. 2000)
Faecal samples	McMasters test	Continuous	Strongyle eggs, Strongyloides eggs, *Coccidia* spp.	(Hansen and Perry, 1994)
	Direct Baermans technique	Binary	*Dictyocaulus viviparous*	(Hansen and Perry, 1994)
	Modified Ziehl Nelsens technique	Binary	*Cryptosporidium* spp.	(Hansen and Perry, 1994)
	Sedimentation	Binary	*Fasciola* spp., *Paramphistoma* spp., *Schistosoma* spp.	(Hansen and Perry, 1994)
	Larval cultures	Binary	*Haemonchus placei, Ostertagia Ostertagi, Trichostrongylus axei, Cooperia* spp., *Bunostomum trigocephalum, Oesophagostomum radiatum, Nematodirus* spp.	(Hansen and Perry, 1994)

### Data analysis

Analysis of growth curves and factors associated with growth rate were carried out using mixed-effects models, which can account for both temporal and spatial correlation in the data. By accounting for the sequential structure in the fixed and random effects and in the correlation structure, it is possible to include time-varying predictors such as infection status directly in the analysis. Their effect is modelled against their occurrence time, enabling insights that would otherwise not be seen by ignoring the sequential structure (Willett, 1997; Gröhn *et al*. 1999). Here, we investigate how the live weight changes with age, and how infection and non-infection factors shape these growth curves. The model used in the analysis is presented in equation (1).(1)
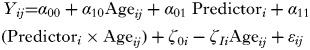
The equation has a structural part and an error part. It models the live body weight of calf *i* at time *j* (*Y*_*ij*_). Using a categorical predictor variable (calf sex) for illustration, the structural part of equation (1) estimates four main parameters of interest corresponding to the fixed effects:

(a) *α*_00_: Estimated initial weight of male calf in the population, which is the reference category – the reference intercept.
(b) *α*_01_: Estimated differential in the initial weight for female calves – the adjusted intercept value for female calves.
(c) *α*_10_: Estimated rate of growth in male calves – the reference slope.
(d) *α*_11_: Estimated differential rate of growth in female calves – the adjusted slope value for female calves.

The statistical significance of these estimated parameters is evaluated to determine if there are significant differences between the starting weights and growth rates among male and female calves. In case the predictor variable is continuous, *α*_00_ would be the estimated initial weight value when the predictor variable *y* is zero, and *α*_11_ would be the differential rate in growth for every unit increase in the level of the predictor variable *Y*. The estimates at time 0 (*j* = 0) may not be interpreted in certain instances dependent on the variable under study (such as estimate of effect of infection at birth when it cannot occur), but generally this does not affect model estimates for the slopes. The continuous variables can be ‘centred’ on their means (by subtracting a constant e.g. the mean value from the predictor before running the model) to facilitate interpretation.

The error part in the composite model equation (1) captures the three sources of random variation in longitudinal studies (Diggle *et al*. 2003):

(a) *Random effects*: (*ζ*_0*i*_) Each study subject has intrinsic characteristics different from those of other subjects in the study, giving each individual a specific response profile. These are incorporated in the models by introducing study subjects as random effects, and modelling the within-individual variation.
(b) *Serial correlation*: (*ζ*_1*i*_Age_*ij*_) Weights recorded from the same individual over time may be correlated, with the correlation between a pair of measurements decreasing with increase in separation time. A number of correlation structures for the repeated measures are considered, and the structure closest to the actual relationship (with the largest log-likelihood among competing models) is selected for use as the base unconditional growth model.
(c) *Measurement error*: (*ε*_*ij*_) The measurement process as taking of live body weights adds variation in the data.

Univariable screening of the putative variables was carried out, and variables with a *P-*value ⩽0·2 offered to the multivariable analysis. Following univariable analysis, interactions between biologically plausible non-infectious factors and infectious factors were tested. Backward elimination was carried out through sequentially removing terms from the maximum model starting with interaction terms and variables with least significant *P*-values. This analysis proceeded in two steps; first with identification of non-infectious factors associated with growth rate, and secondly, while controlling for significant non-infectious factors, determined the relationship between infectious factors and growth rate. Interaction terms were allowed in the final model only if their main effects were significant. The final model contained only significant terms of *P*-value <0·05. The terms were then individually added back and model comparisons made to determine if the terms significantly improved the model fit.

To account for the spatial correlation arising from the 2-stage cluster study design and sampling, sub-location was included as a random effect in the final model. The repeated measures analysis was carried out using *nlme* statistical package (Pinheiro and Bates, 2000), on the R platform (R Development Core Team, 2011). This allows for specification of the correlation structure, and random effects structure. The model in R was coded as follows:
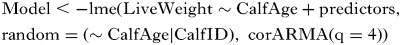
where age of calf (CalfAge) and predictors (e.g. calf sex, infection status etc.) are the fixed effects. Each calf (CalfID) was fitted as a random effect to account for the correlation between measurements taken from the same calf over observation time. An autoregressive-moving average (corARMA) to account for correlations consecutive measurements from the same individual was used. The model diagnostics to check whether distributional assumptions were violated was done by visual inspection of residuals, fitted values and the estimated random effects (Pinheiro and Bates, 2000).

## RESULTS

### Outcome measure

**Fig. 1.**
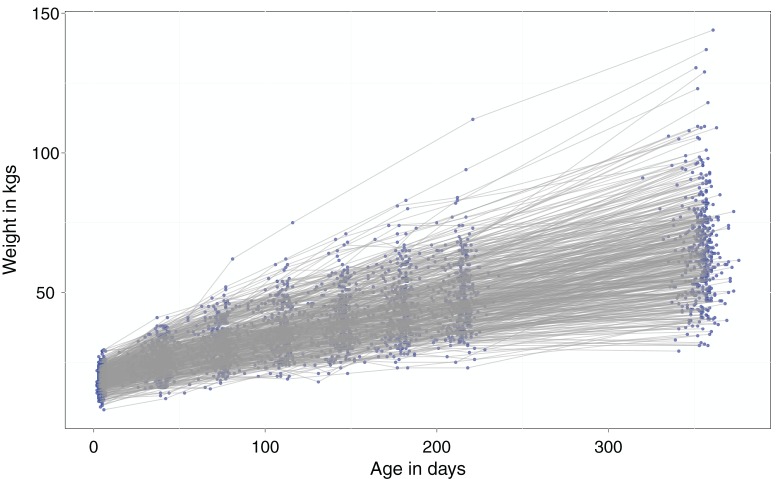
Growth trajectories of the 455 calves that completed the one year observation time. The blue dots are individual's weights recorded and the grey lines connect repeated measures for each calf. Routine weight measurements were done from birth up to week 31 of age, and thereafter at the final visit done at week 51 before leaving the study.

The mean daily weight gain (growth rate) was estimated at 0·134 kg (0·124, 0·146; 95% CI). The mean live body weight at recruitment was 19·2 kg±3·7 SD (range 8–29·5), and at one year 65·2 kg±17·72 SD (range 29–144). A large variation in growth rates of up to 10-fold difference (minimum 0·03 kg and maximum 0·34 kg daily weight gain) was observed. The percentage body weight gain over one year ranged from as low as 50% to as high as over 700%. Variation in live body weight increased with age (see Fig. 1).

### Mixed-effect models

The selection of the growth function to adopt for analysis of factors affecting growth was based on a formal examination of model fit using different growth functions, examination of graphical growth trajectories, and the ease of interpretation of the growth parameters from the models. Based on these criteria, a linear growth model with a varying slope and intercept and assuming a moving average correlation structure was selected as the unconditional growth model. Adding sub-location as a random effect did not significantly improve the model fit. The comparison between the linear models is presented in Supplementary Table 1S – in Online version only. This was subsequently used in the analysis of infectious and non-infectious factors associated with growth rates in zebu calves.

Results from univariable analysis testing the association between infectious and non-infectious factors showing variables with a *P-*value <0·2 and offered to the multivariable analysis are presented in Supplementary Tables 2 and 3S – in Online version only. After model simplification, the final model estimated growth rate at 134·7 g day^−1^ (equivalent to 49·2 kg weight gain in a year), see results of final model in Table 2.

**Table 2.** Results of minimum adequate mixed model showing the significant infectious and non-infectious factors associated with growth rate (kg day^−1^) in zebu calves under one year. Dam heart girth size and farm altitude (elevation) were centred around their mean values to facilitate interpretation

Variable	Estimate	lowerCI	upperCI	DF	*t*-value	*P*-value
Intercept						
Initial weight estimate	20·855	19·344	22·366	1055	27·09	<0·001
(Dam Heart girth size-135)/10	−0·464	−1·403	0·474	1055	−0·97	0·332
Occupation – salaried	−1·230	−3·685	1·225	427	−0·99	0·325
(Elevation – 1240)/100	0·872	−0·456	2·199	427	1·29	0·198
Watering at homestead	0·805	−0·801	2·412	427	0·99	0·325
Calf sex – female	−0·058	−1·654	1·537	427	−0·07	0·943
*T. parva* seropositive	1·109	−0·355	2·574	1055	1·49	0·137
*T. mutans* seropositive	−0·339	−1·637	0·958	1055	−0·51	0·608
*A. marginale* seropositive	2·409	0·248	4·571	1055	2·19	0·029
*Trichophyton* spp.	1·096	−2·254	4·446	1055	0·64	0·521
(Strongyle epg/1000)	0·287	−0·147	0·722	1055	1·3	0·195
Growth rate (slope)						
Age in days	0·134	0·124	0·146	1055	24·28	<0·001
Age:(Dam heart girth−135)/10	0·016	0·010	0·021	1055	5·49	<0·001
Age: Occupation – salaried	0·023	0·008	0·038	1055	2·94	0·003
Age: (Elevation−1240)/100	0·012	0·003	0·020	1055	2·68	0·007
Age: Watering at homestead	0·012	0·001	0·022	1055	2·23	0·026
Age: Calf sex – female	−0·013	−0·023	−0·003	1055	−2·52	0·012
Age: *T. parva* seropositive	−0·018	−0·028	−0·008	1055	−3·62	<0·001
Age: *T. mutans* seropositive	−0·002	−0·012	0·008	1055	−0·41	0·683
Age: *A. marginale* seropositive	−0·002	−0·014	0·010	1055	−0·33	0·741
Age: *Trichophyton* spp.	−0·025	−0·047	−0·004	1055	−2·32	0·021
Age: (Strongyle epg/1000)	−0·004	−0·007	−0·002	1055	−3·38	<0·001
Age: *T. parva–T. mutans*	0·012	0·004	0·019	1055	2·9	0·004
Age: *T. parva–A. marginale*	−0·011	−0·019	−0·003	1055	−2·76	0·006
Intra-class correlation	0·79					

Calves from farms where the farmer had a salaried income had higher growth rates, gaining 8·3 kg more in a year compared with farms where the farmer was not salaried. Female calves gained 4·7 kg less compared with male calves in a year. Large heart girth size in the dams was associated with higher growth rates, with a 10 cm deviation from the mean girth of the population associated with 5·8 kg higher gain in a year from the average. An increase in the altitude of the farm by 100 m was associated with a 4·2 kg higher gain in weight in a year.

Controlling for the effects of non-infectious factors, infections with helminths (strongyle EPG count), with fungi *Trichophyton* spp., and with *Theileria parva* seropositive had a significant negative association with growth rate. Additionally, there was evidence of co-infection interactions differing in size and direction; antagonistic interactions between *T. parva* and *Theileria mutans*, and synergistic interactions between *T. parva* and *Anaplasma marginale*.

Calves that had experienced seropositivity for *T. parva* were estimated to gain 6·7 kg less on average compared with animals that did not sero-convert during the one-year observation time. This is the equivalent of 13·7% decrease in average growth rate associated with *T. parva* seropositivity. The model estimate for the effect of infection with *A. marginale*, while controlling for all other significant predictors, was a marginal decrease in growth (0·7 kg difference in weight gained over one year compared with uninfected animals). However, animals co-infected with *T. parva* and *A. marginale* had an estimated growth rate lower than the combined negative effects of each infection, a synergistic interaction. Calves co-infected with the two were estimated to have gained 11·6 kg less in one year compared with uninfected animals, equivalent to 23·6% less than the average growth rates for uninfected animals.

Co-infections between *T. parva* and *T. mutans* were antagonistic with the effect on growth rates of the more pathogenic *T. parva* infections moderated in the presence of *T. mutans*. Whereas the weight gain of *T. parva* seropositive calves was estimated to reduce by 6·7 kg over a year, animals seropositive for both *T. parva* and *T. mutans* were estimated to have a weight gain only 3·3 kg less that of uninfected animals. This is equivalent to a 6·7% decrease in average growth rate associated with *T. parva–T. mutans* co-infections, approximately half (13·7%) that estimated for a *T. parva*-only infection.

**Fig. 2.**
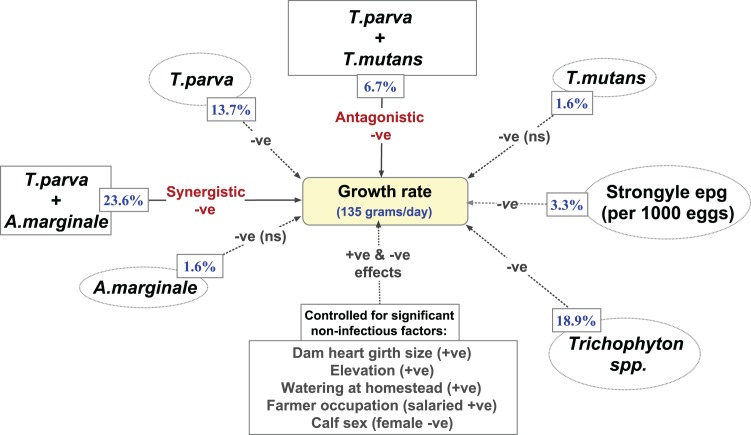
Schematic diagram showing associations between average daily weight gain and different infections and co-infections. Negative associations with ADWG have the sign (−ve), and positive (+ve). All single infections have a negative effect on ADWG. The size of the effect expressed as a percentage of the average growth rate in uninfected animals is shown in blue. Co-infections of *T. parva* and *A. marginale* have a significant negative effect (synergistic) on ADWG, above the sum of their individual effects. Animals co-infected with *T. parva* and *T. mutans* have a significant positive interaction (antagonistic), with average growth rates in coinfected animals higher than in animals infected with *T. parva* only. The model controls for the non-infectious factors. (ns) = non-significant effect.

High worm burden was associated with decreased growth rates. An increase in strongyle epg by a count of 1000 eggs was associated with a 1·6 kg (3·3%) lower gain in the year. Infection with *Trichophyton* spp. was associated with reduced weight gain estimated at 9·3 kg (18·9%) less than the average weight gain in a year. A schematic diagram (Fig. 2) shows the relationship between the daily weight gain (slope in the models) and the significant factors associated with growth rates.

The intra-class correlation (ICC) coefficient was used to determine the fraction of the total residual variation that was accounted for by differences between calves (Weir, 2005). The calculated ICC was 0·79 indicating 79% of the residual variation in growth rates was accounted for by between-calf differences, the remaining being error.

## DISCUSSION

This study has investigated factors that determine growth rates in zebu cattle during the first year of life, specifically assessing the impact of infections and their co-infections on growth rates. In this study growth during the first year of life was best described as a linear growth function, with an estimated growth rate of 134·7 g day^−1^. Similar growth rates (120 g day^−1^) have been reported among zebu calves in Lake Victoria's Rusinga Island, Western Kenya (Latif *et al*. 1995). Although these growth rates were much lower compared with those observed in smallholder farms in parts of Central Kenya (240–290 g day^−1^) mainly keeping improved breeds (Gitau *et al*. 2001), some of the fastest growing animals in the current study gained more than 300 g day^−1^.

The smallholder farms in Central Kenya predominantly keep improved breeds as opposed to those in Western Kenya keeping zebus, but do not themselves meet the recommended 400–500 g day^−1^ target growth rates for dairy farms (Gitau *et al*. 2001; Heinrichs and Radostits, 2001). If the finding that some indigenous zebu cattle are growing at rates higher than improved breeds in smallholder settings has a genetic basis, this points to possible opportunities for improved livestock production through within-breed selection. Although within-breed is considered a slow method of genetic improvement when compared with selection between breeds or cross-breeding, it is more permanent and cumulative in areas of high disease pressures where other breeds easily succumb to disease (FAO, 2007).

While controlling for the important non-infectious factors, this study has identified gastrointestinal worm burden (strongyle epg count), presence of dermatophyte *Trichophyton* spp. and tick-borne disease *T. parva* with its co-infections with *T. mutans* and *A. marginale* to be the important infections associated with greatest negative impact on growth rates.

The impact of helminth infections was a burden-dependent effect with an increase in strongyle epg count by 1000 eggs associated with a 3·3% decrease in growth rate. Some study calves had high helminth burden reaching up to 12 000 epg, which would translate to up to 30% loss in growth rate in heavily infected calves. By colonizing the gastrointestinal tract, helminth infections lead to inefficient feed utilization, and in cases such as infections with hookworms result in pathological lesions on gastrointestinal walls to which they attach. In this study the negative effects of individual worm infections (for example, infections with *Haemonchus placei, Trichostrongylus axei* and *Oesophagostomum radiatum* were all found to negatively affect growth) in the model are masked when strongyle egg counts are introduced as a variable, suggesting strongyle epg may be a good practical composite measure of worm burden in these calves.

Infection with the dermatophyte *Trichophyton* spp., although identified in only 7·8% of the calves, was associated with a large decrease (18·9%) in growth rate. Fungal infections will usually not cause clinical disease or be associated with weight loss but their effect is enhanced in immunosuppressed hosts, a good example being in humans with AIDS. This association is however yet to be confirmed in animals (Blanco and Garcia, 2008). The animals identified infected with *Trichophyton* spp. in this study were systemically affected and with stunted growth. It is likely the fungal infections here may be the result of poor health as opposed to being the cause, and the poor growth observed in infected animals may be related to other underlying conditions.

Regarding tick-borne diseases, seropositivity to *T. parva*, the protozoan parasite causing ECF disease, was associated with the greatest impact on growth; this was estimated as a 13·7% decrease in growth rate compared with *T. parva* seronegative animals. It has previously been reported that indigenous calves infected with *T. parva* had stunted growth even though they did not show any clinical disease (Moll *et al*. 1986). No other tick-borne infection had a significant effect on growth rate as single infections. However, the results suggest pathogen–pathogen interactions in individuals co-infected with *T. parva* and *A. marginale*. The growth rate in animals seropositive for *T. parva* and *A. marginale* had the average daily weight gain reduced by 23·6%, a percentage greater than sum of the effect of individual pathogens (15·3%), suggesting a synergistic interaction (harming the host more).

The processes by which interactions between *T. parva* and *A. marginale* occur are unclear and have not been fully investigated. McHardy and Kiara (1995) observed that about 50% of clinical cases of ECF among improved breeds in Kiambu district, Kenya were complicated by *A. marginale* infections. Experimental studies from their work showed super-infection with *T. parva* resulted in a relapse of severe clinical anaplasmosis and severe anaemia even though *A. marginale* parasitaemia remained low. It may be possible that the effect observed is mediated through the immune system and related to an immune-suppression associated with the destruction of lymphocytes infected with *T. parva*. In the same study, McHardy and Kiara (1995) showed that calves super-infected with *T. parva* when the carrier state of *Anaplasma* had stabilized had mild anaplasmosis disease with a moderate fall in PCV suggesting that ECF may interfere with the immunity to anaplasmosis. Experimental studies looking at immunological changes accompanied by these different infection profiles may help improve understanding of the mechanisms by which *T. parva* and *A. marginale* may be interacting.

Unlike interactions with *A. marginale*, having a *T. parva–T. mutans* co-infection was identified to be advantageous on host growth rate. Animals that were ever seropositive for *T. mutans* had an estimated decrease in growth rate of 6·7%, which was less than half the sum of the estimated *T. parva* and *T. mutans* effects (15·3%), an antagonistic interaction. These data are based on serology results, and data based on presence or absence of parasites in blood, for example Reverse Line Blot across all time points would be useful to confirm results.

This result suggests the presence of *T. mutans*, considered a benign pathogen (Brocklesby *et al*. 1972; Coetzer and Tustin, 2004) may be reducing the negative impact on host growth rate by the more pathogenic *T. parva* infection. The mechanisms by which these two *Theileria* species may be interacting are unclear and may only be postulated. One possibility would be interactions through competition for cell resources, which would occur if the presence of *T. mutans* negatively affected *T. parva* parasite densities, thereby reducing the large impact *T. parva* has on host growth. Such competitive interactions between parasites modifying densities of competing parasites have been demonstrated in other studies (Lello *et al*. 2004; Conlan *et al*. 2009).

The second possible mechanism by which these parasites could interact is through the host immune system. This would happen if immune responses elicited following *T. mutans* infection offers some level of protection against subsequent *T. parva* infections. Currently, there is little evidence in immunology literature supporting this theory and to a large extent solid cross-protection against *Theileria* species is thought to work only among homologous parasites. Immunity against *T. mutans* remains largely unstudied, perhaps because *T. mutans* is normally benign and has attracted little interest among researchers. The protective immunity against *T. parva* is thought to occur in two ways: (a) humoral immunity against the sporozoites injected by infected ticks, and (b) cell-mediated immune responses against macroschizont-infected cells which are thought to express surface antigens that can be targeted by effector killer T-cells. Effects of humoral responses are thought to be limited mainly due to the thousands of sporozoites a single infected tick injects in a host, and the rapidity with which the sporozoites enter target lymphoid cells.

However, *in vitro* studies have demonstrated that antibodies against *T. parva* (Muguga strain) neutralized infectivity not just against homologous sporozoites but against other *T. parva* strains as well (Musoke *et al*. 1984). This finding is important even though it is still unclear how important these humoral responses are in reducing sporozoites infectivity *in vivo*, and whether the observed cross-protection between *T. parva* strains extend to other species such as *T. mutans* or vice versa. This would offer a possible explanation to the observed beneficial effects of a *T. parva* co-infection with *T. mutans*. Experimental work looking at the immune responses with different combinations of the infections with these two *Theileria* species may help improve our understanding of these interactions.

Different ticks transmit the two *Theileria* species: *T. parva* by *Rhipicephalus appendiculatus* and *T. mutans* by *Amblyomma variegatum*. These two ticks share large geographical overlaps in their distribution in East, Central and Southern Africa (Walker *et al*. 2003). This is supported by results of sero-surveillance studies done in different regions showing similar prevalence rates for both *T. mutans* and *T. parva* (Ogden *et al*. 2005; Swai *et al*. 2009; Gachohi *et al*. 2010). This widespread co-occurrence of the two *Theileria* species may indicate that although *T. parva* is still associated with huge losses in livestock, the effect may be moderated to an extent by co-occurrence with *T. mutans*.

The results obtained here identify simple farm management practices that would help improve the growth rates of zebu calves. Although it is not entirely clear how providing drinking water to the animals from within the homestead works, this simple husbandry practice is estimated to be associated with preventing an estimated 8·6% reduction in growth rate compared with farms where animals walk a distance away from the homestead to access water. Secondly, since dam heart girth sizes were identified as good predictors for growth rate in calves, farmers or breeders can improve their decision-making in selecting animals to keep for breeding based on the relative dam sizes.

The two main infections, with high prevalences and strongly associated with decreased growth rate, are helminths and *T. parva* infections. Although animals are infected with many different species of worms, this study has identified strongyle epg count as a good composite measure quantifying the effect helminths have on the host. Data on strongyle epg are relatively easy and inexpensive to collect under field conditions. A herd's helminth burden can be estimated and a decision on helminth control made based on the results. Here, the results show helminth control would prevent the loss of growth rates estimated at up to 30% in animals heavily infected.

Tick control would be expected not only to reduce the direct effects exerted by feeding ticks [the tick *A. variegatum* has been associated with decreased growth rates (Stachurski *et al*. 1993)] but also on the impact of pathogens they transmit. Specifically, it would be expected the beneficial effect would be not just reducing impact of *T. parva* but of the more harmful *T. parva–A. marginale* co-infections. The finding of *T. mutans* reducing the impact of the more pathogenic *T. parva* in cases of co-infections with the two parasites may be a relationship that can be exploited to reduce impacts of *T. parva* infections. Such relationships have been used to control for *Anaplasmosis* where the more benign *Anaplasma centrale* has been used as a vaccine for the more pathogenic *A. marginale* (Kocan *et al*. 2010).

This information points to evidence that by reducing the prevalence of one pathogen, the benefit is likely greater beyond that estimated by just removing the effect of individual pathogens. There is a need to better understand the mechanisms by which *T. parva* interacts with *A. marginale* and with *T. mutans*, possibly through experimental work, as these provide opportunities for improved design of disease-control strategies and increased livestock production.

## Supplementary material

To view supplementary material for this article, please visit http://dx.doi.org/10.1017/S0031182013001261

## Supplementary Material

Supplementary Material Supplementary information supplied by authors.

Supplementary Material Supplementary information supplied by authors.

Supplementary Material Supplementary information supplied by authors.

Supplementary Material Supplementary information supplied by authors.

Supplementary Material Supplementary information supplied by authors.
