# Supplementary material for: Parasite co-infections show synergistic and antagonistic interactions on growth performance of East African zebu cattle under one year
Source: Parasitology. 2013 Sep 4;140(14):1789–98. doi: 10.1017/S0031182013001261 (PMC3829697; doi:10.1017/S0031182013001261)
Supplement: Supplementary Material — Supplementary information supplied by authors. [file S0031182013001261sup001.pdf]

Supplementary Table 1: Comparison between linear models with different correlation structures. Model E was chosen as the best unconditional growth model.

Model	DF	AIC	BIC	logLik	test	L.Ratio	*p*-value
Model A	6	21174.23	21211.07	-10581.12			
Model B	7	21176.23	21219.21	-10581.12	A vs B	0	1
Model C	8	20727	20776.12	-10355.5	B vs C	451.24	< 0.001
Model D	9	20665.75	20721.01	-10323.87	C vs D	63.25	< 0.001
Model E	10	20622.63	20684.03	-10301.31	D vs E	45.12	< 0.001
Model F	11	20622.1	20689.64	-10300.05	E vs F	2.53	0.112

Model A. lme(fixed = Weight ~age, random = (~age |CalfID))

Model B. lme(fixed = Weight ~age, random = (~age |CalfID), correlation = corAR1()).

Model C. lme(fixed = Weight ~age, random = (~age |CalfID), correlation = corARMA(q = 2)).

Model D. lme(fixed = Weight ~ age, random = (~age |CalfID), correlation = corARMA(q = 3)).

Model E. lme(fixed = Weight ~age, random = (~age |CalfID), correlation = corARMA(q = 4)).

Model F. lme(fixed = Weight ~age, random = list(~1 | Sublocation, ~age | CalfID), correlation = corARMA(q = 4)).

corAR1() = autoregressive correlation structure.

corARMA(q) = moving average correlation structure.
