# Supplementary material for: Parasite co-infections show synergistic and antagonistic interactions on growth performance of East African zebu cattle under one year
Source: Parasitology. 2013 Sep 4;140(14):1789–98. doi: 10.1017/S0031182013001261 (PMC3829697; doi:10.1017/S0031182013001261)
Supplement: Supplementary Material — Supplementary information supplied by authors. [file S0031182013001261sup002.pdf]

Supplementary Table 2: Results of univariable analysis showing the (slope) and non-infectious factors, using mixed models.

Variable	estimate	Std.Error	DF	t-value	p-value
Farmer's sex- male	0.0051	0.0048	2972	1.0659	0.2865
Farmer's age	-0.0003	0.0002	2946	-1.803	0.0715
Education-primary school	0.0005	0.0065	2952	0.0695	0.9446
Education-secondary school	0.0044	0.0075	2952	0.5833	0.5597
Occupation-salaried	0.0098	0.0065	2953	1.4951	0.135
Tropical livestock units	-0.0011	0.0003	2966	-3.0823	0.0021
Total acres owned	-0.0001	0.0004	2803	-0.173	0.8627
Watering at homestead	0.011	0.0045	2902	2.4352	0.0149
Distance to water	0.009	0.0053	2958	1.7084	0.0877
Housing calves	0.0047	0.0074	1716	0.6399	0.5223
Suckling	0.0267	0.005	2966	5.3261	0.001
Graze with adults	0.008	0.0094	2928	0.8462	0.3975
Milk prior calving	-0.0051	0.0076	2941	-0.6751	0.4997
Milk post calving	0.0058	0.0054	2966	1.0776	0.2813
Use supplements	-0.0013	0.0059	2954	-0.2262	0.8211
Vaccine use	0.0177	0.0108	2972	1.6367	0.1018
Veterinary support	-0.0074	0.0061	2952	-1.2208	0.2222
Knowledge of diseases	-0.005	0.0054	2953	-0.922	0.3566
Housing - stall-shed	-0.0062	0.0046	2952	-1.3578	0.1746
Tick control -yes	0.0026	0.0051	2972	0.5011	0.6163
Trypanosome control - yes	0.001	0.0063	2972	0.1559	0.8762
Worm control - yes	-0.0096	0.0049	2972	-1.9525	0.051
Antibiotics use - yes	0.0026	0.0045	2972	0.5707	0.5682
Moderate introgression	0.0083	0.0065	2964	1.2868	0.1983
Substantial introgression	0.0277	0.0106	2964	2.6225	0.0088

Heterozygosity	0.331	0.1225	2905	2.7022	0.0069
Calf sex	-0.0064	0.0044	2972	-1.4506	0.147
Recruitment weight	0.0024	0.0006	2972	4.1339	<0.001
Dam heart girth size	0.0013	0.0002	2319	6.9765	<0.001
Dam - antibodies *T.parva*	-0.0001	0.0001	2319	-0.0813	0.9357
Dam - antibodies *T.mutans*	-0.0001	0.0001	2319	-0.5489	0.5866
Dam - antibodies *A.marginale*	-0.0002	0.0002	2319	-1.5673	0.3717
Dam - antibodies *B.bigemina*	-0.0002	0.0001	2319	-0.0002	0.1203
Body condition score - dam	0.0056	0.0012	2328	4.8449	<0.001
Mean monthly NDVI	-0.0196	0.0108	2971	-1.8071	0.0709
Rhipicephalus appendiculatus	-0.0125	0.0031	2971	-4.0364	< 0.001
Amblyomma variegatium	-0.0226	0.0017	2971	-13.5362	< 0.001
Boophilus microplus	-0.0039	0.0031	2971	-1.2597	0.2079
Rhipicephalus evertsi	-0.0149	0.0022	2971	-6.6807	< 0.001
Lice	-0.025	0.0034	2971	-7.3473	< 0.001
Fleas	0.004	0.0042	2970	0.9593	0.3375
