# Supplementary material for: Parasite co-infections show synergistic and antagonistic interactions on growth performance of East African zebu cattle under one year
Source: Parasitology. 2013 Sep 4;140(14):1789–98. doi: 10.1017/S0031182013001261 (PMC3829697; doi:10.1017/S0031182013001261)
Supplement: Supplementary Material — Supplementary information supplied by authors. [file S0031182013001261sup003.pdf]

Supplementary Table 3: Results of mixed models univariable analysis of infectious factors and their interactions associated with growth rate (slope).

Variable	estimate	Std.Error	DF	t-value	p-value
Anaplasma spp. - microscopy	-0.0107	0.0087	2971	-1.2332	0.2176
Babesia spp. - microscopy	-0.0986	0.0545	2971	-1.8081	0.0707
Theileria spp. - microscopy	-0.0119	0.0017	2971	-7.1119	<0.001
Trypanosoma spp. - microscopy	-0.0137	0.0074	2971	-1.834	0.0668
Trypanosoma vivax - microscopy	-0.0155	0.0102	2971	-1.5146	0.13
Trypanosoma theileri - microscopy	0.0082	0.0321	2971	0.2564	0.7977
T.parva - serology	-0.0171	0.0013	2972	-13	<0.001
T.mutans - serology	-0.0099	0.0018	2972	-5.6048	<0.001
A.marginale - serology	-0.0072	0.0017	2972	-4.2699	<0.001
B.bigemina - serology	-0.0095	0.002	2972	-4.7441	<0.001
T.parva:T.mutans	0.0066	0.0025	2970	2.6208	0.0088
T.parva:A.marginale	-0.0063	0.0027	2970	-2.3652	0.0181
T.parva:B.bigemina	0.0005	0.0033	2970	0.1523	0.879
T.mutans:A.marginale	0.0017	0.0038	2970	0.444	0.6571
B.bigemina:A.marginale	0.003	0.0036	2970	0.8363	0.4031
Calicophoron spp.	-0.0141	0.0024	2971	-5.9688	<0.001
Coccidia spp.	-0.0094	0.0019	2971	-4.9724	<0.001

Cooperia spp.	0.0055	0.0211	2971	0.2626	0.7929
Dictyocaulus viviparus	-0.0114	0.0049	2971	-2.3389	0.0194
Fasciola spp.	-0.0153	0.007	2971	-2.1812	0.0293
Haemonchus placei	-0.0148	0.0016	2971	-9.0233	<0.001
Microfilaria spp.	0.0065	0.0209	2971	0.311	0.7558
Moniezia spp.	-0.8659	0.4184	2971	-2.0695	0.0386
Nematodirus spp.	0.0304	0.0357	2971	0.8514	0.3946
Oesophagostomum radratium	-0.014	0.0032	2971	-4.4257	<0.001
Ostertagia ostertagi	-0.0185	0.0405	2971	-0.4577	0.6472
Strongyle epg/1000	-0.0046	0.0012	1565	-4.0208	<0.001
Strongyloides spp.	-0.0105	0.003	2971	-3.5214	<0.001
Toxocara vitulorum	0.0025	0.0063	2971	0.3961	0.692
Trichophyton spp.	-0.0291	0.0059	2971	-4.9206	<0.001
Trichostrongylus axei	-0.0151	0.0021	2971	-7.2579	<0.001
Trichuris spp.	-0.0202	0.0084	2971	-2.3955	0.0167
