# Supplementary figures and images for: Parasite co-infections show synergistic and antagonistic interactions on growth performance of East African zebu cattle under one year
Source: Parasitology. 2013 Sep 4;140(14):1789–98. doi: 10.1017/S0031182013001261 (PMC3829697; doi:10.1017/S0031182013001261)

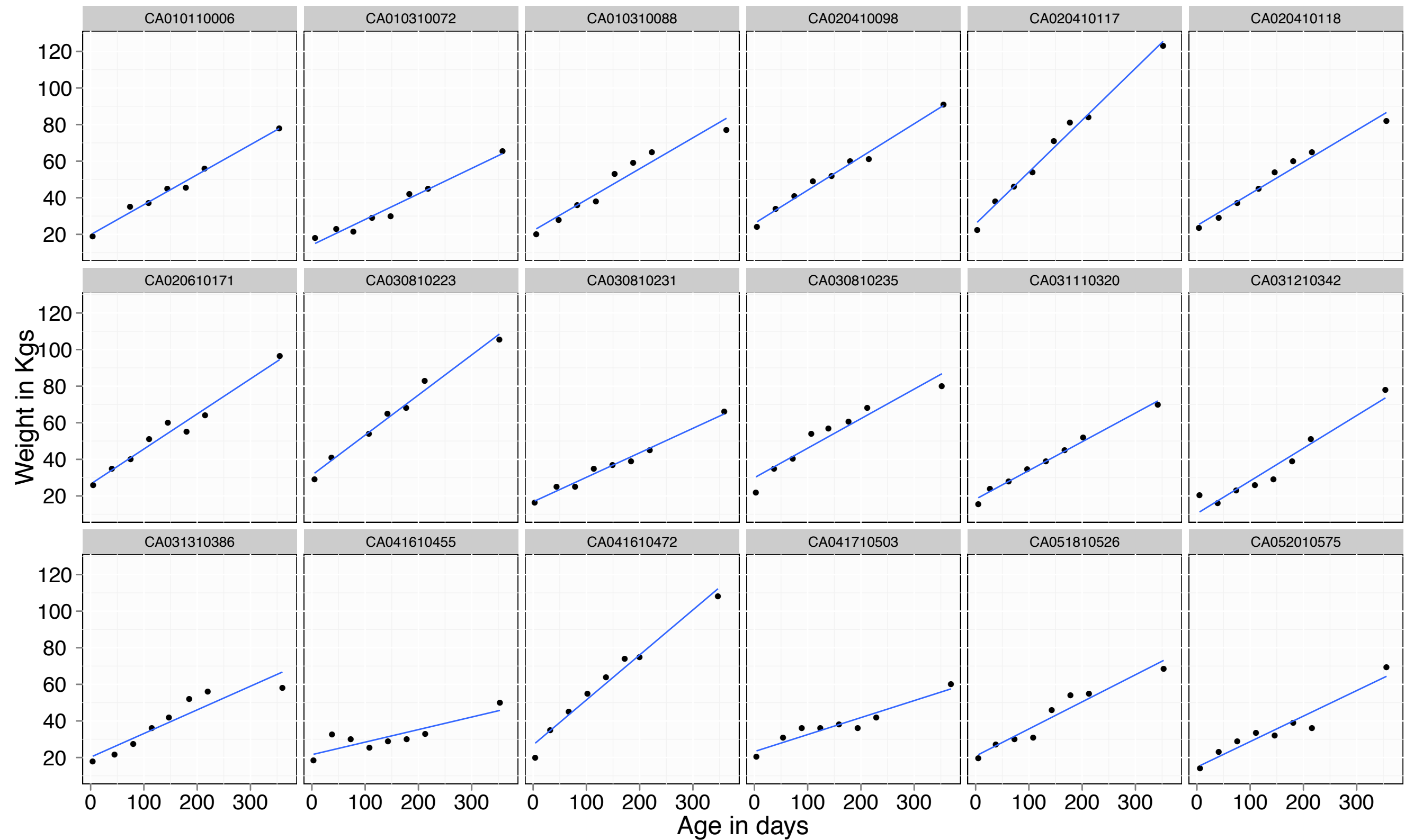

Supplement: Supplementary Material — Supplementary information supplied by authors. [file S0031182013001261sup004.pdf]

**(A) Model Structure (I)**

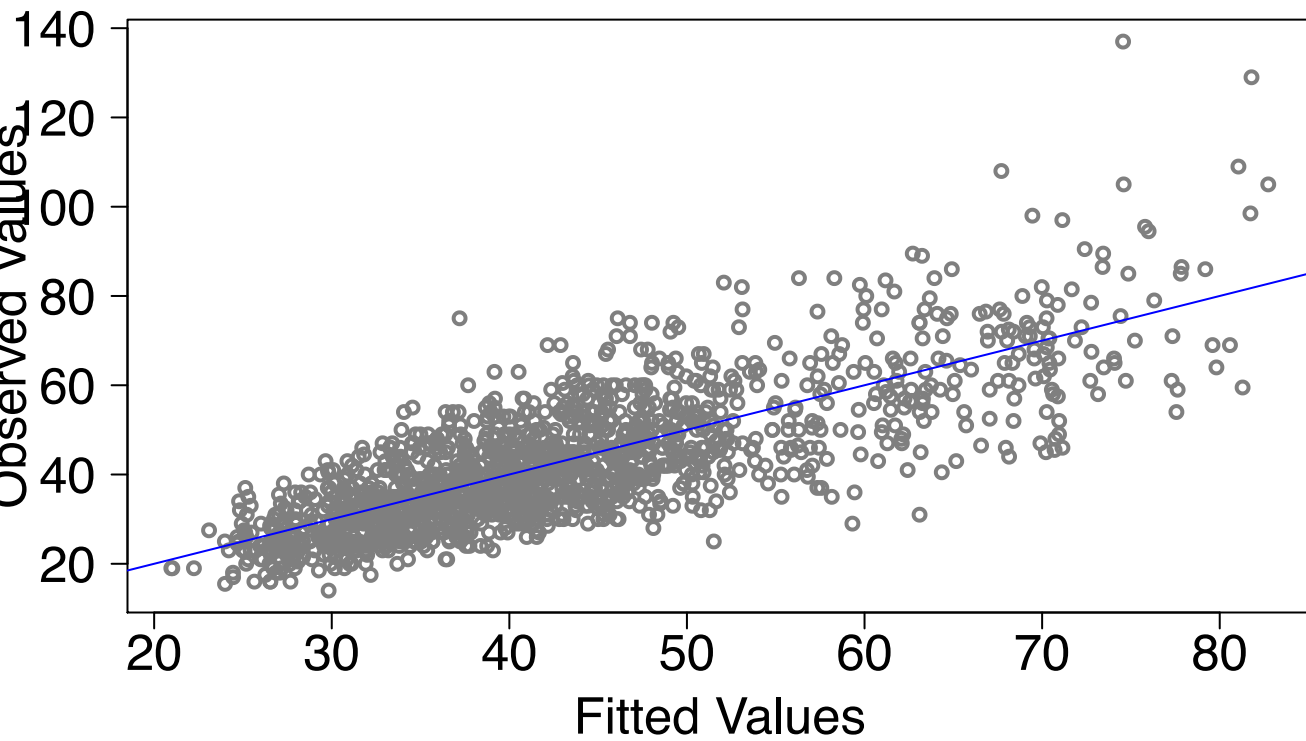

**(B) Model Structure (II)**

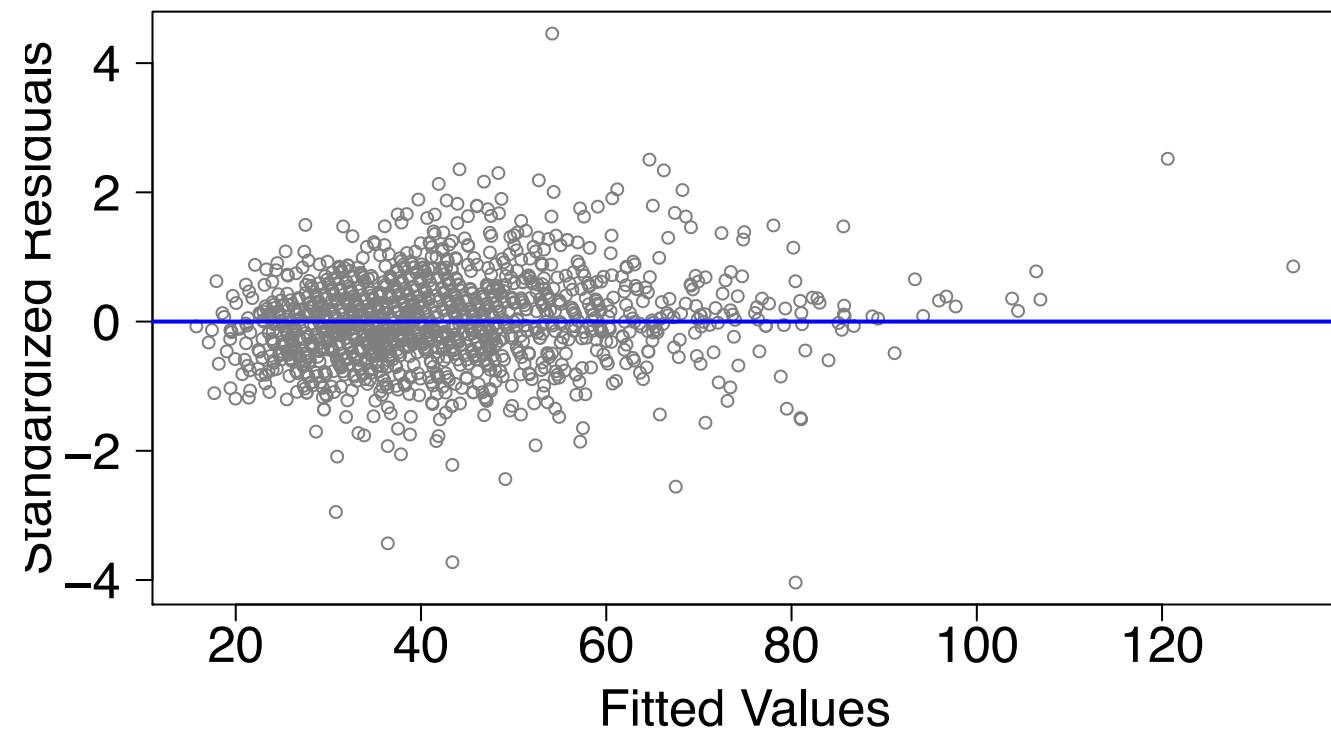

**(C) Q-Q Normal – Group Random Effects**

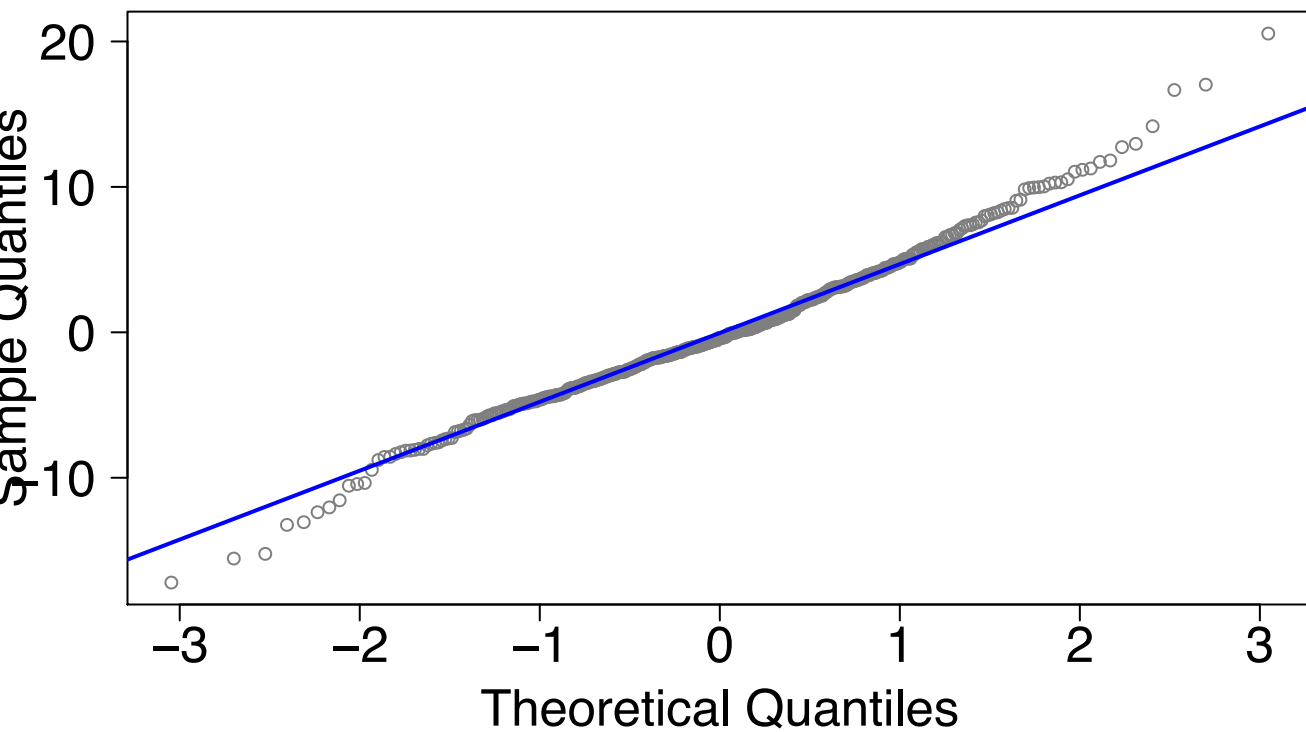

**(D) Q-Q Normal – Residuals**

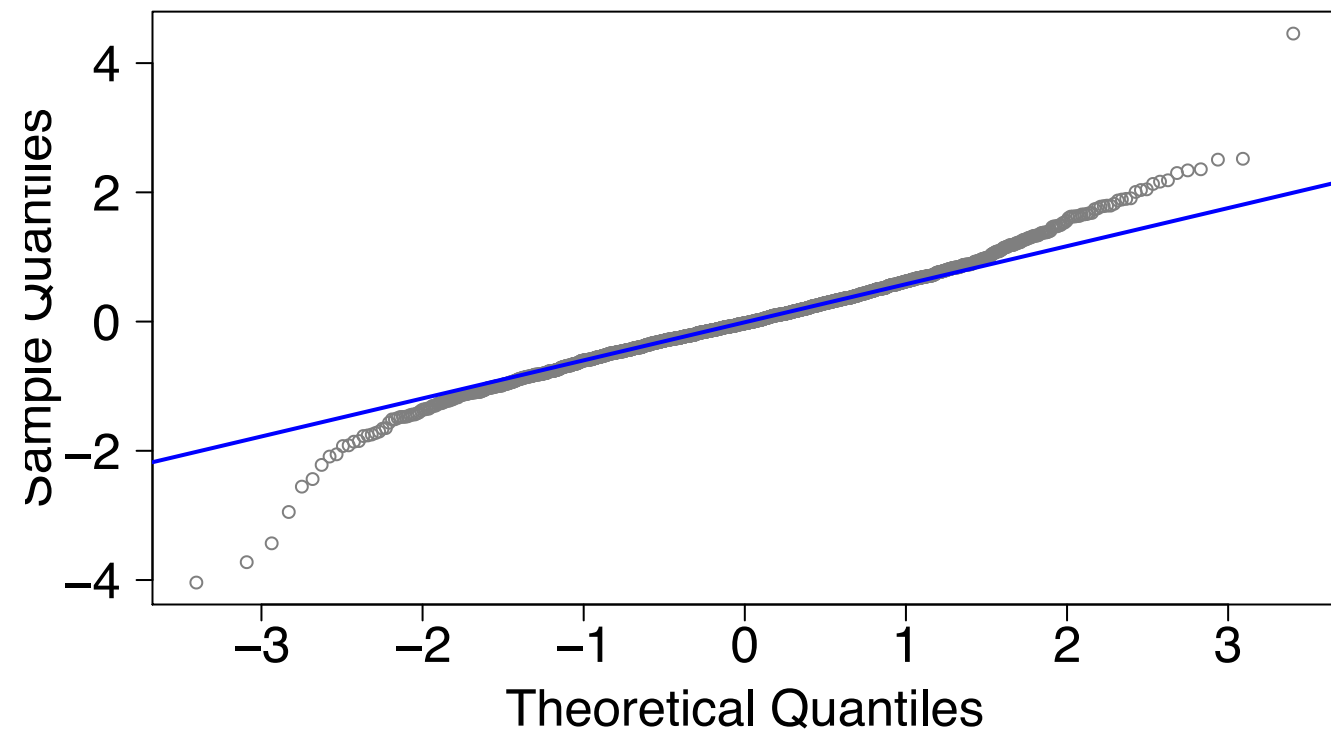

Supplement: Supplementary Material — Supplementary information supplied by authors. [file S0031182013001261sup005.pdf]
